# Supplementary material for: Long-COVID symptom monitoring: Insights from a two-year telemedicine study
Source: PLoS One. 2024 Jul 26;19(7):e0307834. doi: 10.1371/journal.pone.0307834 (PMC11280159; doi:10.1371/journal.pone.0307834)

PLOS ONE

Monitoring Long-COVID Symptoms at a Two-Year Follow-Up Through a Telemedicine Platform

--Manuscript Draft--

Manuscript Number:	PONE-D-24-17457
Article Type:	Research Article
Full Title:	Monitoring Long-COVID Symptoms at a Two-Year Follow-Up Through a Telemedicine Platform
Short Title:	Long-COVID at a Two-Year Follow-Up Through Telemedicine
Corresponding Author:	VALERIA CALCATERRA<br>University of Pavia: Universita degli Studi di Pavia<br>Pavia, ITALY
Keywords:	monitoring, Long-COVID, long-term, telemedicine, telemonitoring
Abstract:	Background: The heterogeneous manifestations of Long-COVID have become increasingly important, significantly impacting patients' lives. Telemedicine is an important tool for the follow-up post COVID-19. This study is a follow-up to a part of a large cohort study involving COVID-positive patients at the COD19 telemedicine platform operations center. We recontacted patients who were initially monitored from February 2020 to May 2020 to investigate the presence of Long-COVID symptoms at a 2-year follow-up.<br>Methods: We conducted a 2-year follow-up interview on Long-COVID symptoms and explored whether patients had contracted a second COVID-19 infection between the 1-year and 2-year follow-ups, as well as their vaccination status.<br>Results: Of the 165 patients studied, 139 (84%) reported symptoms at the 1-year follow-up, while only 101 (61%) reported symptoms at the 2-year follow-up. Among patients with Long-COVID symptoms at the 2-year follow-up, the majority (80, 49%) had Long-COVID at the 1-year follow-up, received the SARS-CoV-2 vaccine, and had not experienced a second infection between the two follow-ups. Both having Long-COVID at the 1-year follow-up and contracting a second infection were significant risk factors for presenting with Long-COVID at the 2-year follow-up.<br>Conclusions: To the best of our knowledge, this study stands out as one of the few that includes a 2-year follow-up on Long-COVID symptoms using telemedicine. Telemedicine has proven to be an innovative tool for monitoring patients over an extended period effectively and for early diagnosis and treatment of conditions. Telemedicine represents a future health challenge.
Order of Authors:	Andrea Foppiani
	Chiara Montanari
	Sara Zanelli
	Michele Davide Maria Lombardo
	VALERIA CALCATERRA
	Gianvincenzo Zuccotti
Additional Information:	
Question	Response
**Financial Disclosure**<br><br>Enter a financial disclosure statement that describes the sources of funding for the work included in this submission. Review the submission guidelines for detailed requirements. View published research	The author(s) received no specific funding for this work.

articles from *PLOS ONE* for specific examples.<br><br>This statement is required for submission and **will appear in the published article** if the submission is accepted. Please make sure it is accurate.<br><br>**Funded studies**<br>Enter a statement with the following details:<br>• Initials of the authors who received each award<br>• Grant numbers awarded to each author<br>• The full name of each funder<br>• URL of each funder website<br>• Did the sponsors or funders play any role in the study design, data collection and analysis, decision to publish, or preparation of the manuscript?<br><br>Did you receive funding for this work?	
**Competing Interests**<br><br>Use the instructions below to enter a competing interest statement for this submission. On behalf of all authors, disclose any competing interests that could be perceived to bias this work—acknowledging all financial support and any other relevant financial or non-financial competing interests.<br><br>This statement is required for submission and **will appear in the published article** if the submission is accepted. Please make sure it is accurate and that any funding sources listed in your Funding Information later in the submission form are also declared in your Financial Disclosure statement.<br><br>View published research articles from *PLOS ONE* for specific examples.	The authors have declared that no competing interests exist.

**NO authors have competing interests**<br><br>Enter: *The authors have declared that no competing interests exist.*<br><br>**Authors with competing interests**<br><br>Enter competing interest details beginning with this statement:<br><br>*I have read the journal's policy and the authors of this manuscript have the following competing interests: [insert competing interests here]*<br><br>* typeset	
**Ethics Statement**<br><br>Enter an ethics statement for this submission. This statement is required if the study involved:<br><br>• Human participants<br>• Human specimens or tissue<br>• Vertebrate animals or cephalopods<br>• Vertebrate embryos or tissues<br>• Field research<br><br>Write "N/A" if the submission does not require an ethics statement.<br><br>General guidance is provided below. Consult the submission guidelines for detailed instructions. **Make sure that all information entered here is included in the Methods section of the manuscript.**	The study was conducted according to the guidelines of the Declaration of Helsinki, and approved by the Ethics Commission (Milano Area 1 number: n. 2023/ST/023, protocol 0017685/2023). Written informed consent was obtained from all participants.

**Format for specific study types**<br><br>**Human Subject Research (involving human participants and/or tissue)**<br>• Give the name of the institutional review board or ethics committee that approved the study<br>• Include the approval number and/or a statement indicating approval of this research<br>• Indicate the form of consent obtained (written/oral) or the reason that consent was not obtained (e.g. the data were analyzed anonymously)<br><br>**Animal Research (involving vertebrate animals, embryos or tissues)**<br>• Provide the name of the Institutional Animal Care and Use Committee (IACUC) or other relevant ethics board that reviewed the study protocol, and indicate whether they approved this research or granted a formal waiver of ethical approval<br>• Include an approval number if one was obtained<br>• If the study involved *non-human primates*, add *additional details* about animal welfare and steps taken to ameliorate suffering<br>• If anesthesia, euthanasia, or any kind of animal sacrifice is part of the study, include briefly which substances and/or methods were applied<br><br>**Field Research**<br><br>Include the following details if this study involves the collection of plant, animal, or other materials from a natural setting:<br>• Field permit number<br>• Name of the institution or relevant body that granted permission	
**Data Availability**<br><br>Authors are required to make all data underlying the findings described fully available, without restriction, and from the time of publication. PLOS allows rare exceptions to address legal and ethical concerns. See the PLOS Data Policy and FAQ for detailed information.	Yes - all data are fully available without restriction

A Data Availability Statement describing where the data can be found is required at submission. Your answers to this question constitute the Data Availability Statement and **will be published in the article**, if accepted.<br><br>**Important:** Stating 'data available on request from the author' is not sufficient. If your data are only available upon request, select 'No' for the first question and explain your exceptional situation in the text box.<br><br>Do the authors confirm that all data underlying the findings described in their manuscript are fully available without restriction?	
**Describe where the data may be found in full sentences. If you are copying our sample text, replace any instances of XXX with the appropriate details.**<br><br>• If the data are **held or will be held in a public repository**, include URLs, accession numbers or DOIs. If this information will only be available after acceptance, indicate this by ticking the box below. For example: *All XXX files are available from the XXX database (accession number(s) XXX, XXX.).*<br>• If the data are all contained **within the manuscript and/or Supporting Information files**, enter the following: *All relevant data are within the manuscript and its Supporting Information files.*<br>• If neither of these applies but you are able to provide **details of access elsewhere**, with or without limitations, please do so. For example:<br><br>*Data cannot be shared publicly because of [XXX]. Data are available from the XXX Institutional Data Access / Ethics Committee (contact via XXX) for researchers who meet the criteria for access to confidential data.*<br><br>*The data underlying the results presented in the study are available from (include the name of the third party*	All relevant data are within the manuscript and its Supporting Information files.

and contact information or URL).<br>• This text is appropriate if the data are owned by a third party and authors do not have permission to share the data.<br><br>* typeset	
Additional data availability information:	

Manuscript

Click here to access/download;Manuscript;LongCovid Telemed_01_05_24 text.docx

Monitoring Long-COVID Symptoms at a Two-Year Follow-Up Through a Telemedicine Platform

Short title: Long-COVID at a Two-Year Follow-Up Through Telemedicine

[1,2]Andrea Foppiani^, [2,3]Chiara Montanari^, [4]Sara Zanelli, [5]Michele Davide Maria Lombardo, [6,4]Valeria Calcaterra*, [3,4]Gianvincenzo Zuccotti

^equal contribution

[1]International Center for the Assessment of Nutritional Status (ICANS), Department of Food, Environmental and Nutritional Sciences (DeFENS), University of Milan, 20133 Milan, Italy.

[2]IRCCS Istituto Auxologico Italiano, Clinical Nutrition Unit, Department of Endocrine and Metabolic Medicine, 20100 Milan, Italy

[3]Department of Biomedical and Clinical Science, University of Milan, 20157 Milan, Italy

[4]Pediatric Department, "V. Buzzi" Children's Hospital, 20154 Milan, Italy

[5]Orthopaedics and Traumatology, University of Milan, 20157 Milan, Italy

[6]Department of Internal Medicine, University of Pavia, 27100 Pavia, Italy

***Corresponding author:**

Valeria Calcaterra

Department of Internal Medicine University of Pavia and

Pediatric Department, Buzzi Children's Hospital,

Via Castelvetro n. 32, 20154 Milano, Italy,

valeria.calcaterra@unipv.it

Abstract

Background: The heterogeneous manifestations of Long-COVID have become increasingly important, significantly impacting patients' lives. Telemedicine is an important tool for the follow-up post COVID-19. This study is a follow-up to a part of a large cohort study involving COVID-positive patients at the COD19 telemedicine platform operations center. We recontacted patients who were initially monitored from February 2020 to May 2020 to investigate the presence of Long-COVID symptoms at a 2-year follow-up.

Methods: We conducted a 2-year follow-up interview on Long-COVID symptoms and explored whether patients had contracted a second COVID-19 infection between the 1-year and 2-year follow-ups, as well as their vaccination status.

Results: Of the 165 patients studied, 139 (84%) reported symptoms at the 1-year follow-up, while only 101 (61%) reported symptoms at the 2-year follow-up. Among patients with Long-COVID symptoms at the 2-year follow-up, the majority (80, 49%) had Long-COVID at the 1-year follow-up, received the SARS-CoV-2 vaccine, and had not experienced a second infection between the two follow-ups. Both having Long-COVID at the 1-year follow-up and contracting a second infection were significant risk factors for presenting with Long-COVID at the 2-year follow-up.

Conclusions: To the best of our knowledge, this study stands out as one of the few that includes a 2-year follow-up on Long-COVID symptoms using telemedicine. Telemedicine has proven to be an innovative tool for monitoring patients over an extended period effectively and for early diagnosis and treatment of conditions. Telemedicine represents a future health challenge.

Key words: monitoring, Long-COVID, long-term, telemedicine, telemonitoring

Introduction

Since 2020, COVID-19 has affected millions of people around the world. In addition to acute disease, SARS-CoV-2 could determine a condition called Long-COVID characterized by long lasting symptoms. Long-COVID is an emerging disorder frequently underestimated and undertreated, but its heterogenous manifestations have become increasingly important with a significant impact on patients' lives.

The World Health Organization (WHO) defined Long-COVID as the persistence or development of new symptoms 3 months after the acute infection, with these symptoms lasting for at least 2 months without any possible explanation [1]. Afterwards, in 2022 the National Institute for Health and Care Excellence (NICE) described Long-COVID as sign and symptoms that persist or appear following acute infection. In NICE's guidelines were defined two conditions: ongoing symptomatic COVID-19, which lasts from 4 to 12 weeks, and post COVID-19 syndrome, lasting 12 weeks or more [2].

Overall, the incidence of Long-COVID is estimated in at least 10% of SARS-CoV-2 infections [3]. Long-COVID occurrence is not related to the severity of COVID-19: asymptomatic or patients with mild acute disease could develop this syndrome even if the risk is lower. The pathophysiology of Long-COVID is still not clarified. Several hypotheses have been postulated, in particular persistent virus presence, autoimmunity, microbiome dysbiosis and tissue damage are some of these [4]. Further studies are needed to better understand this topic and define consequently specific treatments.

Long-COVID is a complex multisystemic condition involving cardiovascular, respiratory, gastrointestinal, endocrinological, neuropsychiatric, musculoskeletal, dermatological and genitourinary systems. Fatigue, dyspnea, cognitive disorders (e.g., brain fog), myalgia, joint pain and depression are the most common reported symptoms in adult population. The variety of symptoms of this syndrome could be explained by the widespread expression of angiotensin converting enzyme 2 (ACE2) receptor, which is the specific gateway for SARS-CoV-2 entry, in the epithelium of many organs [5]. It is important to highlight that there are no specific treatments for this syndrome up to date. Some interventions seem to have a positive impact in particular physical activity with targeted exercises, nutritional support, symptomatic medications

(e.g., non-steroidal anti-inflammatory drugs) and psychological support. Vaccination could indirectly reduce the probability of developing Long-COVID reducing the risk of SARS-CoV-2 infection [6].

The impact of Long-COVID on the quality of life and daily functioning is noteworthy, therefore is essential monitoring patients after the acute infection to early detect symptoms of Long-COVID and manage them through a multidisciplinary approach.

Telemedicine is an important tool for the follow-up post COVID-19. During pandemic, we proposed a telemedicine platform for an active surveillance service for home-care patients. Initially it was a project a project of the University of Milan - Faculty of Medicine, in collaboration with ATS of Milan and ASST Fatebenefratelli-Sacco, designed during the COVID-19 emergency to provide an active home surveillance service [7]. The name was COD19 ("discharged operation center"), a virtual "ad hoc" platform, and the aim was monitoring and managing clinical symptoms, isolation verification, and general need status of discharged patients as well as healthcare workers with COVID-19 infection. The service was expanded to cover Milan's major public hospitals and, during the second wave, COD19 further permitted to follow patients tested positive for COVID19, not necessarily hospitalized, with the aim to monitor patients at higher risk of complications in the acute phase of the disease. COD19 experience has shown the potential of telemedicine and evolved into a full virtual hospital called "COD20 - Home Hospital Care", where a patient-specialist video consultation service was possible [7].

COD20 represents a virtual hospital model, created to ensure continuity of care for patients at home through technological solutions. This digital platform was subsequently utilized to monitor the progression of the disease in patient management.

Telemedicine is a healthcare application modality that aims not to replace traditional medicine but to integrate it. It enables remote healthcare delivery with the goal of reducing hospital admissions or anticipating discharge through close monitoring of the patient's health status [8]. This is applicable in cases of acute diseases, such as COVID-19, where patients may require surveillance during acute infection or post-discharge to detect warning symptoms and signs and reduce the risk of complications or flare-ups.

Telemedicine can also be enhanced by the provision of data derived from sensors (e.g., oxygen saturation), a practice known as telemonitoring [9].

Moreover, telemedicine finds application as a part of a diagnostic pathway or during follow-up of chronic patients, improving the quality of care and assistance, especially of chronic patients [10].

Thus, telemedicine is considered as an integral part of the services of the National Health Service, according to the recent guidelines (2020) by the Italian Ministry of Health, allowing for costs optimization and with a psycho-social impact [8].

This study is a follow-up to a part of a large cohort study involving COVID-positive patients at the telemedicine platform COD19 operations center. We followed up with patients who were initially monitored from February 2020 to May 2020 and later recontacted to investigate the presence of Long-COVID symptoms at a 2-year follow-up. The objective of this report is to share our experience and highlight the potential value of telemedicine platforms in epidemic situations and other conditions where isolation is necessary.

Methods

Study Design and Patient's Selection

We originally included all patients with a confirmed diagnosis of COVID-19, which was performed by molecular swab and polymerase chain reaction positive for viral ribonucleic acid. All patients were discharged by the monitoring service after reaching clinical stability and after performing 2 negative SARS-CoV-2 swabs within 24 hours of each other. All patients that were included in the 1-year follow-up study were also recontacted for this 2-year follow-up study.

We originally excluded the following categories of patients: patients who died during the follow-up or after discharge; patients hospitalized at follow-up; patients under the age of 18; patients with psychiatric disorders; patients who refused to participate in the study; and patients who could not be contacted.

Eligible patients were consecutively contacted, and, after expressing consent to participate in the study, a semi structured interview was administered by phone interview between February and March 2022 by trained medical staff from the COD19 operations center [7].

The study was conducted according to the guidelines of the Declaration of Helsinki, and was approved by both the Ethics Commission of the University of Milan for patient enrollment during the pandemic (protocol number 126/20) and the Ethics Commission Milano Area 1 for long-term monitoring (number 2023/ST/023, protocol 0017685/2023). Written informed consent was obtained from all participants.

Semi structured Interview

We based the 2-year follow-up interview on the results from our previous study. The most frequent symptoms reported in the literature were sought. A total of 37 items were surveyed and grouped into the following categories: respiratory disorders, fatigue and weakness, muscle and joint pain, movement impairments, neurological and cognitive impairments, sensory alterations, sleep disorders, and gastrointestinal symptoms.

We also explored whether the patient contracted a second COVID-19 infection between the 1-year and the 2-year follow-up, and the vaccination status.

All data regarding the acute phase of the disease were collected during monitoring by the COD19 operation center and either retrieved from electronic clinical records or by directly interviewing the patient when information was unavailable. Data included date of birth, sex, body mass index (BMI), smoking habits, symptoms at onset, admission to the hospital, type of hospitalization (in ward or in intensive care unit [ICU]), medications taken during hospitalization and after hospital discharge (corticosteroids, antivirals, antibiotics, anticoagulants), and comorbidities (hypertension, diabetes, cardiovascular diseases, malignant tumors, chronic obstructive pulmonary disease, and chronic kidney disease).

Disease severity during the acute phase, and eventually the second infection, was graded in 7 levels according to Huang et al. [11]: (1) discharged from the emergency room, asymptomatic or with mild symptoms; (2) discharged from the emergency room, with symptoms; (3) hospitalized, not requiring

supplemental oxygen; (4) hospitalized, requiring supplemental oxygen; (5) hospitalized, requiring high-flow nasal cannula, noninvasive mechanical ventilation (NIV), or both; (6) admitted to hospital requiring extracorporeal membrane oxygenation, invasive mechanical ventilation, or both; (7) death. Categories of BMI were defined as follows: underweight, BMI < 18.5 kg/m²; normal weight, BMI 18.5–24.9 kg/m²; overweight, BMI 25.0–29.9 kg/m²; obese, BMI ≥30 kg/m².

Statistical Analysis

Continuous variables are reported as median and interquartile range (IQR) (25th, 75th percentile), whereas categorical variables are reported as count (fraction).

Participants were categorized into 2 groups according to the Huang et al. scale [10]: 1–2 categories (not hospitalized with or without symptoms) and 3–6 categories (hospitalized, requiring or not oxygen or admitted to ICU).

Multivariable logistic generalized additive models were used to describe the relationship between symptoms at 2-year follow-up (dichotomous, present/not present) and age (continuous), sex (dichotomous, female/male), acute phase severity (dichotomous, scale 1–2/scale 3–6), symptoms at 1-year follow-up (dichotomous, present/not present), second infection (dichotomous, infected/not infected), and vaccination status (dichotomous, vaccinated/not vaccinated). Linearity between outcomes and age was not assumed by using a smooth term with a thin replate regression spline. Statistical analyses were performed in R 4.3.3.

Results

Three hundred three 303 patients originally included in the 1-year follow-up study were consecutively contacted between February and March 2022. Of those, 165 were included in the 2-year follow-up study, and patient characteristics are presented in Table 1.

Sexes were almost equally distributed (53% females, 47% males), and median age was 53 (IQR, 42–62) years. Most patients had a normal weight, with a median BMI of 24.9 Kg/m^2 (IQR, 22.6–28.0). Although

most patients never smoked (59%), the most prevalent comorbidities were hypertension (28%), cardiovascular diseases (9.1%), and diabetes (7.9%).

Almost all patients (97%) were vaccinated at the 2-year follow-up, but only 80% received 3 doses of the vaccine.

Only 10% of patients contracted a second SARS-CoV-2 infection, and their severity was generally low, never exceeding scale 2 of the acute phase severity scale.

Table 1. Patients characteristics at the acute infection, vaccination status, and second infection occurrence.

**Characteristic**	**N = 165**[1]
Sex	
Female	88 (53%)
Male	77 (47%)
Age (years)	53 (42, 62)
Body mass index (kg/m²)	24.9 (22.6, 28.0)
Body mass index category	
Underweight	6 (3.6%)
Normal weight	79 (48%)
Overweight	55 (33%)
Obese	25 (15%)
Smoking habits	
Never smoked	98 (59%)
Ex-smoker	41 (25%)
Smoker	26 (16%)
Hypertension	47 (28%)
Diabetes	13 (7.9%)
Cardiovascular disease	15 (9.1%)
Cerebrovascular disease	4 (2.4%)
Malignant tumor	9 (5.5%)
Chronic obstructive pulmonary disease	6 (3.6%)
Chronic kidney disease	1 (0.6%)
Acute phase severity	
Scale 1: Not hospitalized, asymptomatic	6 (3.6%)
Scale 2: Not hospitalized, symptomatic	63 (38%)

**Characteristic**	**N = 165**[1]
Scale 3: Hospitalized, not requiring oxygen	21 (13%)
Scale 4: Hospitalized, requiring oxygen (nasal cannula)	37 (22%)
Scale 5: Hospitalized, requiring oxygen (NIV)	36 (22%)
Scale 6: Hospitalized, ICU	2 (1.2%)
Corticosteroid	5 (3.0%)
Antivirals	100 (61%)
Antibiotics	67 (41%)
Heparin	49 (30%)
Received COVID-19 vaccine	160 (97%)
Completed doses of COVID-19 vaccine	
0	5 (3.0%)
1	1 (0.6%)
2	27 (16%)
3	132 (80%)
Second infection	17 (10%)
Second infection severity	
None	148 (90%)
Scale 1: Not hospitalized, asymptomatic	1 (0.6%)
Scale 2: Not hospitalized, symptomatic	16 (9.7%)
Scale 3: Hospitalized, not requiring oxygen	0 (0%)
Scale 4: Hospitalized, requiring oxygen (nasal cannula)	0 (0%)
Scale 5: Hospitalized, requiring oxygen (NIV)	0 (0%)
Scale 6: Hospitalized, ICU	0 (0%)

[1]n (%); Median (IQR)

Of the 165 patients studied, 139 (84%) reported symptoms at 1-year follow-up, while only 101 (61%) reported symptoms at 2-year follow-up.

Figure 1 shows how categories of Long-COVID at 1-year follow-up and at 2-year follow-up evolve based on vaccination and second infection status.

Of patients with Long-COVID symptoms at 2-year follow-up, most patients (80, 49%) had Long-COVID at 1-year follow-up, received the SARS-CoV-2 vaccine, and had not experienced a second infection between the 2 follow-ups.

Figure 1. Alluvial plot showing prevalence of Long-COVID at 1- and 2-year after acute infection, stratified by vaccination status and second infection occurrence.

Table 2 and Table 3 show the results from logistic generalized additive models of presenting Long-COVID at 2-years follow-up.

Of the parameters studied, both having Long-COVID at 1-year follow-up and contracting a second infection results in significant risk factors for presenting Long-COVID at 2-years follow-up. This was true also for subdomain of symptoms presented in Table 3, although with some exceptions: sleep disorders did not present those risk factors, and the second infection was significant only in some subdomains.

Interestingly, vaccination did not result in a protective factor, although very few patients in our sample did not receive vaccination.

Table 2. Logistic generalized additive model of any symptom of Long-COVID at 2-year after the acute infection.

**Characteristic**	**log (OR)**[1]	**95% CI**[1]	**p-value**
Had symptoms 1 year after acute infection	2.3	1.2, 3.5	**<0.001**
Sex			
Female	—	—	
Male	-0.02	-0.73, 0.70	>0.9
Acute phase severity			
Scale 1-2: Not hospitalized	—	—	
Scale 3-6: Hospitalized	-0.26	-1.1, 0.55	0.5
Second infection	2.3	0.47, 4.1	**0.013**
Received COVID-19 vaccine	-0.54	-2.8, 1.8	0.6
Age smooth[2]			0.072

[1]OR = Odds Ratio, CI = Confidence Interval

[2]Smooth term by low rank isotropic smoothers of age in years

Table 3. Logistic generalized additive model of specific symptom domains of Long-COVID at 2-year after the acute infection.

**Characteristic**	**Neurological and cognitive impairments**			**Fatigue and weakness**			**Movement impairments**			**Muscle and joint pain**			**Respiratory disorders**			**Sensory alterations**			**Sleep disorders**		
	Log (OR)[1]	95% CI[1]	p-value	Log (OR)[1]	95% CI[1]	p-value	Log (OR)[1]	95% CI[1]	p-value	Log (OR)[1]	95% CI[1]	p-value	Log (OR)[1]	95% CI[1]	p-value	Log (OR)[1]	95% CI[1]	p-value	Log (OR)[1]	95% CI[1]	p-value
**Had symptoms 1 year after acute infection**	1.2	0.43, 2.1	**0.003**	1.9	1.1, 2.8	**<0.001**	1.7	0.64, 2.8	**0.002**	2.0	1.1, 2.9	**<0.001**	1.7	0.87, 2.5	**<0.001**	2.7	1.5, 3.8	**<0.001**	0.69	-0.10, 1.5	0.087
**Sex**																					
Female	—	—		—	—		—	—		—	—		—	—		—	—		—	—	
Male	0.24	-0.57, 1.0	0.6	-0.23	-0.97, 0.52	0.5	-0.40	-1.5, 0.66	0.5	0.32	-0.47, 1.1	0.4	-0.44	-1.3, 0.38	0.3	-0.17	-1.1, 0.74	0.7	-0.52	-1.3, 0.26	0.2
**Acute phase severity**																					
Scale 1-2: Not hospitalized	—	—		—	—		—	—		—	—		—	—		—	—		—	—	
Scale 3-6: Hospitalized	-0.43	-1.3, 0.43	0.3	0.25	-0.60, 1.1	0.6	0.59	-0.67, 1.8	0.4	-0.65	-1.5, 0.22	0.14	0.80	-0.19, 1.8	0.11	-0.38	-1.4, 0.63	0.5	0.14	-0.72, 0.99	0.8
**Second infection**	0.60	-0.61, 1.8	0.3	1.2	-0.04, 2.5	0.057	1.0	-0.55, 2.6	0.2	1.7	0.53, 3.0	**0.005**	2.7	1.3, 4.2	**<0.001**	2.2	0.75, 3.6	**0.003**	0.18	-1.1, 1.4	0.8
**Received COVID-19 vaccine**	-1.2	-3.3, 0.91	0.3	0.85	-1.5, 3.2	0.5	-0.85	-3.3, 1.6	0.5	0.65	-1.7, 3.0	0.6	43	-58,822,389,	>0.9	-1.6	-3.7, 0.52	0.14	0.24	-2.0, 2.5	0.8

Characteristic	Neurological and cognitive impairments			Fatigue and weakness			Movement impairments			Muscle and joint pain			Respiratory disorders			Sensory alterations			Sleep disorders		
	Log (OR)[1]	95% CI[1]	p-value	Log (OR)[1]	95% CI[1]	p-value	Log (OR)[1]	95% CI[1]	p-value	Log (OR)[1]	95% CI[1]	p-value	Log (OR)[1]	95% CI[1]	p-value	Log (OR)[1]	95% CI[1]	p-value	Log (OR)[1]	95% CI[1]	p-value
														58,822,475							
**Age smooth[2]**			0.093			0.12			0.2			0.9			0.2			0.8			>0.9

[1]OR = Odds Ratio, CI = Confidence Interval

[2]Smooth term by low rank isotropic smoothers of age in years

Discussion

During the COVID emergency, telemedicine has been essential because hospitals and communities care service were saturated, COVID-19 patients were isolated at home and also the rest of the population to contain the spread of pandemic. Furthermore, telemedicine has also proven to be useful in monitoring long lasting symptoms after SARS-CoV-2 infection [8]. These long-term consequences of COVID-19 are an emerging issue with a detrimental impact on patients' functioning and overall quality of life.

Thus, our telephone surveillance can be considered as an operative mode of telemedicine that allowed remote monitoring of the health status of patients with past SARS-CoV2 acute infection, therefore considered at risk of developing long-COVID. More generally, the use of telemedicine makes possible an "home health care" through a remote follow-up over time. In case of long-COVID development, telemedicine meets multiple health needs, providing continuity and equity in access to health care [10].

To the best of our knowledge, this study stands out as one of the few that includes a 2-year follow-up on Long-COVID symptoms. We found that 84% of the patients exhibited symptoms one year after the acute infection. However, this number decreased to 61% after a 2-year follow-up. Although reinfections were not common during the follow-up period, we did observe a higher risk of Long-COVID symptoms among patients who experienced a second infection within the 2-year timeframe.

Evidences on Long-COVID following SARS-CoV-2 reinfections are still limited. According to our study the persistence of symptoms post COVID-19 is more frequent after the first infection than after the subsequent ones. Moreover, as reported mainly in older patients, the cumulative risk of Long-COVID is higher proportionally to the number of SARS-CoV-2 infections. Nevertheless, it is important to note that older patients often have pre-existing comorbidities and may experience a more severe acute infection, which could further increase their risk [12].

COVID-19 severity is associated to the risk of developing Long-COVID. Critical disease, acute respiratory distress syndrome, long-term ventilator support and multiorgan impairment caused by COVID-19 are considered as risk factors for persistence of symptoms [4]. A severe COVID-19 which is associated to ICU admission and hospitalization, is described by Tsampasian et al. as a risk factor for Long-COVID [13]. Nevertheless, Long-COVID is described also in non-hospitalized patients with mild acute disease [14],

although the frequency is lower than in severe acute SARS-CoV-2 infections. In our population SARS-CoV-2 infection are mild, symptomatic but hospitalization was not necessary.

The risk of Long-COVID is also influenced by SARS-CoV-2 variants [15]. Specifically, the risk appears to decrease with the spread of the Omicron variant [4]. Additionally, reinfections have become more common following the emergence of the Omicron variant, and their frequency has progressively increased [9].

Vaccination and anti-SARS-CoV-2 immunity is protective against Long-COVID and this seems to suggest that reinfections with SARS-CoV-2 could reduce the probability of diagnosing Long-COVID [15]. Several studies have clearly demonstrated the protective effect of vaccination against the onset of Long-COVID [12]. In our study, however, we did not find this positive association. It's worth noting that the number of unvaccinated patients in our sample was significantly small.

Our findings highlight no gender difference in Long-COVID onset, although according to several studies the female sex is more frequently affected by persistent symptoms post-acute infection [16,17]. According to the results of this study, COVID-19 seems to be more frequent in people with chronic comorbidities such as hypertension, diabetes and cardiovascular diseases as suggested by the scientific literature [18]. Lifestyle can also affect the risk of long lasting symptoms: current smokers have a higher risk compared to those who have never smoked [16]. Smoking may contribute to the persistence of symptoms because of compromised immune and cardiovascular systems [19]. This study could not confirm this association because most patients were non-smokers. Similarly, in our cohort of patients the presence of obesity and overweight are not associated with high disease risk.

More than 50% of patients reported symptoms of Long-COVID at 2-year follow-up. Long-COVID can have a heterogeneous clinical presentation, involving respiratory symptoms, muscular and joint complains, neurological and sleep disorders and gastrointestinal symptoms, with a burden on daily life of patients. These data underline the importance of a long-term follow-up of these patients in the post-acute infection period, since the high prevalence of Long-COVID symptoms onset, to guarantee a multidisciplinary patient care, or a specialized referral in case of a specific disorder. A specific intervention of rehabilitation may be necessary [20]. A remote surveillance can be considered an effective, simple, and well-accepted follow-up modality of telemedicine, in order to offer a long-term health care of patients with previous COVID19 and to ameliorate their quality of life

The sample size of our study is small, thus further research on a wider cohort and with a longer follow-up are needed. Telemedicine has some barriers to its use, for example accessibility, the costs to instrument maintenance and insufficient legal regulations, but they are not insurmountable obstacles given its usefulness in monitoring many clinical conditions.

Conclusion

Through the COD19 platform, we have monitored patients post-COVID-19 for two years, still identifying the persistence of Long-COVID symptoms. The potential value of telemedicine platforms is evident in pandemic situations and in other conditions where isolation is essential. Telemedicine has proven to be an innovative tool for effectively monitoring patients over an extended period and for early diagnosis of conditions that require treatment. It represents a future challenge in healthcare

Author Contributions: Conceptualization, A.F., C.M., V.C., G.V.; formal analysis A.F., M.D.M.L.; investigation, A.F., C.M., M.D.M.L., S.Z.; writing—original draft preparation, A.F., C.M., S.Z., M.D.M.L., V.C.; writing—review and editing, A.F., C.M., V.C., G.V.; supervision, A.F., C.M., V.C., G.V.; funding acquisition, G.Z. All authors have read and agreed to the published version of the manuscript.

Ethical approval: The study was conducted according to the guidelines of the Declaration of Helsinki, and was approved by both the Ethics Commission of the University of Milan for patient enrollment during the pandemic (protocol number 126/20) and the Ethics Commission Milano Area 1 for long-term monitoring (number 2023/ST/023, protocol 0017685/2023). Written informed consent was obtained from all participants.

Availability of data and materials: All data are incorporated into the article and its online supplementary material.

Competing interests: The authors declare that they have no competing interests

Funding: The project received contributions from (1) Bando Cariplo Networking research & training post-COVID protocol number 2021–4490; (2) HORIZON-HLTH-2021-CORONA-01 CoVICIS project number 101046041; (3) PRIN: research projects of significant national interest - 2022 call prot. 20228pnnjl.

Acknowledgments: not applicable

References

1. World Health Organization (WHO). (2022). *Post COVID-19 condition (Long COVID)*. https://www.who.int/europe/news-room/fact-sheets/item/post-covid-19-condition

2. NICE. (2023). COVID-19 rapid guideline: Managing COVID-19. *National Institute for Health and Care Excellence (NICE): London, UK*.

3. Davis HE, McCorkell L, Vogel JM, Topol EJ. Long COVID: major findings, mechanisms and recommendations. Nat Rev Microbiol. 2023 Mar;21(3):133-146. doi: 10.1038/s41579-022-00846-2. Epub 2023 Jan 13. Erratum in: Nat Rev Microbiol. 2023 Jun;21(6):408. PMID: 36639608; PMCID: PMC9839201.

4. Su S, Zhao Y, Zeng N, Liu X, Zheng Y, Sun J, Zhong Y, Wu S, Ni S, Gong Y, Zhang Z, Gao N, Yuan K, Yan W, Shi L, Ravindran AV, Kosten T, Shi J, Bao Y, Lu L. Epidemiology, clinical presentation, pathophysiology, and management of long COVID: an update. Mol Psychiatry. 2023 Oct;28(10):4056-4069. doi: 10.1038/s41380-023-02171-3. Epub 2023 Jul 25. PMID: 37491461.

5. Nalbandian A, Sehgal K, Gupta A, Madhavan MV, McGroder C, Stevens JS, Cook JR, Nordvig AS, Shalev D, Sehrawat TS, Ahluwalia N, Bikdeli B, Dietz D, Der-Nigoghossian C, Liyanage-Don N, Rosner GF, Bernstein EJ, Mohan S, Beckley AA, Seres DS, Choueiri TK, Uriel N, Ausiello JC, Accili D, Freedberg DE, Baldwin M, Schwartz A, Brodie D, Garcia CK, Elkind MSV, Connors JM, Bilezikian JP, Landry DW, Wan EY. Post-acute COVID-19 syndrome. Nat Med. 2021 Apr;27(4):601-615. doi: 10.1038/s41591-021-01283-z. Epub 2021 Mar 22. PMID: 33753937; PMCID: PMC8893149.

6. Watanabe A, Iwagami M, Yasuhara J, Takagi H, Kuno T. Protective effect of COVID-19 vaccination against long COVID syndrome: A systematic review and meta-analysis. Vaccine. 2023 Mar 10;41(11):1783-1790. doi: 10.1016/j.vaccine.2023.02.008. Epub 2023 Feb 8. PMID: 36774332; PMCID: PMC9905096.

7. Zuccotti GV, Bertoli S, Foppiani A, Verduci E, Battezzati A. COD19 and COD20: An Italian Experience of Active Home Surveillance in COVID-19 Patients. Int J Environ Res Public Health. 2020 Sep 14;17(18):6699. doi: 10.3390/ijerph17186699. PMID: 32938009; PMCID: PMC7560013.

8. Zuccotti G, Calcaterra V, Foppiani A. Present and future of telemedicine for pediatric care: an Italian

regional experience. Ital J Pediatr. 2023 Jan 17;49(1):10. doi: 10.1186/s13052-023-01408-9. PMID: 36650538; PMCID: PMC9843098.

9. Indicazioni Nazionali per l'erogazione Di Prestazioni in Telemedicina, Accordo Stato Regioni ai sensi Art.4 comma 1 D. Lgs. 28 agosto 1997, n.281 (2020). https://www.statoregioni.it/it/conferenza-stato-regioni/sedute-2020/seduta-del-17122020/atti/repertorio-atto-n-215csr/

10. Zuccotti G, Calcaterra V. Telemedicine and E-Health: An Innovative Challenge in Pediatric Care. Int J Environ Res Public Health. 2023 Jan 23;20(3):2091. doi: 10.3390/ijerph20032091. PMID: 36767457; PMCID: PMC9915758.

11. Huang C, Huang L, Wang Y, Li X, Ren L, Gu X, Kang L, Guo L, Liu M, Zhou X, Luo J, Huang Z, Tu S, Zhao Y, Chen L, Xu D, Li Y, Li C, Peng L, Li Y, Xie W, Cui D, Shang L, Fan G, Xu J, Wang G, Wang Y, Zhong J, Wang C, Wang J, Zhang D, Cao B. 6-month consequences of COVID-19 in patients discharged from hospital: a cohort study. Lancet. 2023 Jun 17;401(10393):e21-e33. doi: 10.1016/S0140-6736(23)00810-3. Epub 2023 Jun 12. PMID: 37321233; PMCID: PMC10258565.

12. Bowe B, Xie Y, Al-Aly Z. Acute and postacute sequelae associated with SARS-CoV-2 reinfection. Nat Med. 2022 Nov;28(11):2398-2405. doi: 10.1038/s41591-022-02051-3. Epub 2022 Nov 10. PMID: 36357676; PMCID: PMC9671810.

13. Tsampasian V, Elghazaly H, Chattopadhyay R, Debski M, Naing TKP, Garg P, Clark A, Ntatsaki E, Vassiliou VS. Risk Factors Associated With Post-COVID-19 Condition: A Systematic Review and Meta-analysis. JAMA Intern Med. 2023 Jun 1;183(6):566-580. doi: 10.1001/jamainternmed.2023.0750. PMID: 36951832; PMCID: PMC10037203.

14. Bai F, Tomasoni D, Falcinella C, Barbanotti D, Castoldi R, Mulè G, Augello M, Mondatore D, Allegrini M, Cona A, Tesoro D, Tagliaferri G, Viganò O, Suardi E, Tincati C, Beringheli T, Varisco B, Battistini CL, Piscopo K, Vegni E, Tavelli A, Terzoni S, Marchetti G, Monforte AD. Female gender is associated with long COVID syndrome: a prospective cohort study. Clin Microbiol Infect. 2022 Apr;28(4):611.e9-611.e16. doi: 10.1016/j.cmi.2021.11.002. Epub 2021 Nov 9. PMID: 34763058; PMCID: PMC8575536.

15. Krishna B, Wills M, Sithole N. Long COVID: what is known and what gaps need to be addressed. Br Med Bull. 2023 Sep 12;147(1):6-19. doi: 10.1093/bmb/ldad016. PMID: 37434326; PMCID: PMC10502447.

16. Subramanian A, Nirantharakumar K, Hughes S, Myles P, Williams T, Gokhale KM, Taverner T, Chandan JS, Brown K, Simms-Williams N, Shah AD, Singh M, Kidy F, Okoth K, Hotham R, Bashir N, Cockburn N, Lee SI, Turner GM, Gkoutos GV, Aiyegbusi OL, McMullan C, Denniston AK, Sapey E, Lord JM, Wraith DC, Leggett E, Iles C, Marshall T, Price MJ, Marwaha S, Davies EH, Jackson LJ, Matthews KL, Camaradou J, Calvert M, Haroon S. Symptoms and risk factors for long COVID in non-hospitalized adults. Nat Med. 2022 Aug;28(8):1706-1714. doi: 10.1038/s41591-022-01909-w. Epub 2022 Jul 25. PMID: 35879616; PMCID: PMC9388369.

17. Yong SJ. Long COVID or post-COVID-19 syndrome: putative pathophysiology, risk factors, and treatments. Infect Dis (Lond). 2021 Oct;53(10):737-754. doi: 10.1080/23744235.2021.1924397. Epub 2021 May 22. PMID: 34024217; PMCID: PMC8146298.

18. Martono, Fatmawati F, Mulyanti S. Risk Factors Associated with the Severity of COVID-19. Malays J Med Sci. 2023 Jun;30(3):84-92. doi: 10.21315/mjms2023.30.3.7. Epub 2023 Jun 27. PMID: 37425387; PMCID: PMC10325129.

19. Wong MC, Huang J, Wong YY, Wong GL, Yip TC, Chan RN, Chau SW, Ng SC, Wing YK, Chan FK. Epidemiology, Symptomatology, and Risk Factors for Long COVID Symptoms: Population-Based, Multicenter Study. JMIR Public Health Surveill. 2023 Mar 7;9:e42315. doi: 10.2196/42315. PMID: 36645453; PMCID: PMC9994465.

20. Calcaterra V, Tagi VM, D'Auria E, Lai A, Zanelli S, Montanari C, Biganzoli EM, Marano G, Borghi E, Massa V, Riva A, Zuccotti G. Long-term effects of SARS-CoV-2 infection in hospitalized children: findings from an Italian single-center study. Ital J Pediatr. 2024 Feb 14;50(1):27. doi: 10.1186/s13052-024-01596-y. PMID: 38355648; PMCID: PMC10865522.

Figure 1

Click here to access/download;Figure;Figure 1.png

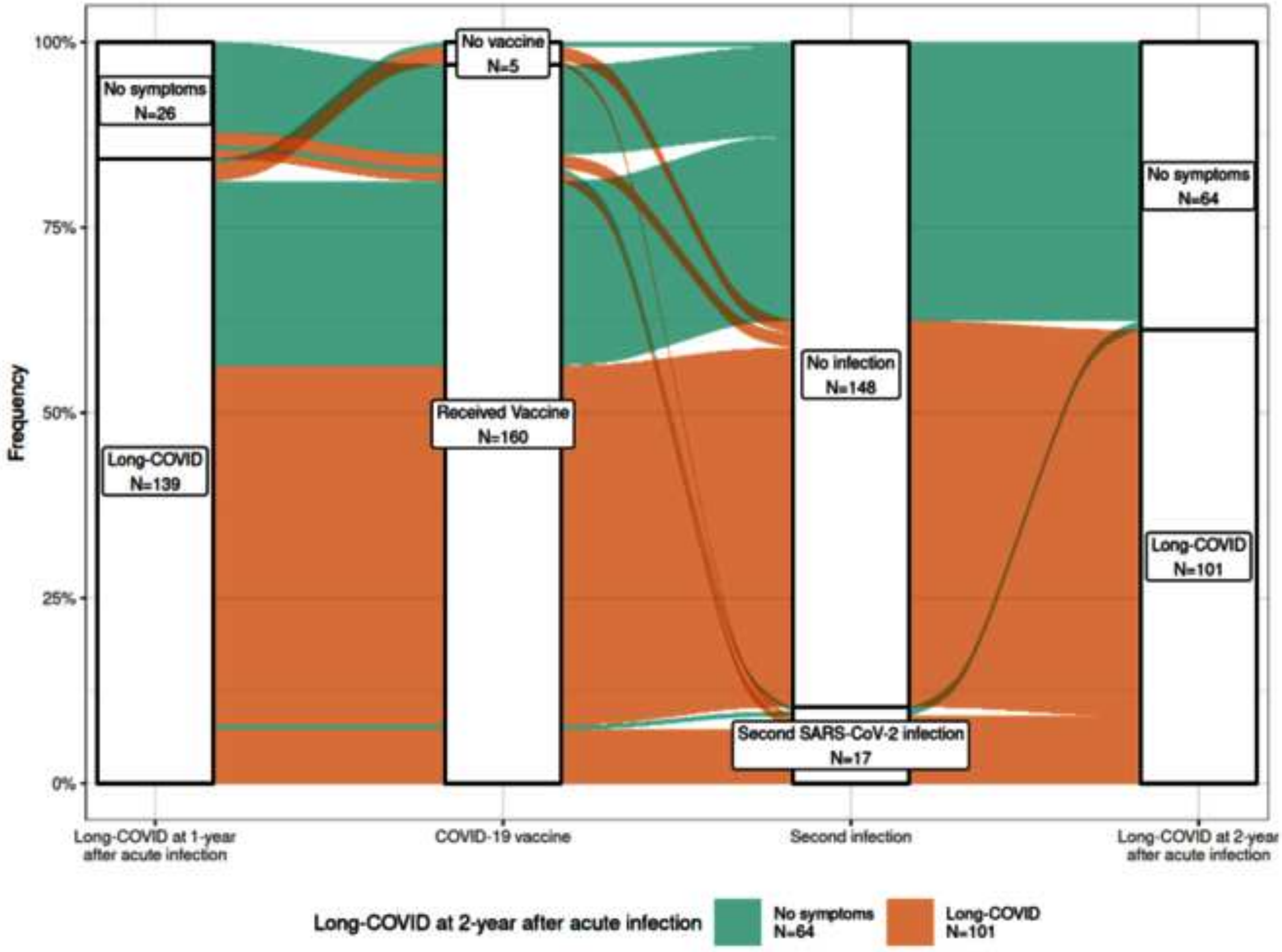

Supplement: Attachment — Submitted filename: PONE-D-24-17457_reviewer_Opuni.pdf [file pone.0307834.s001.pdf]
